# Feeding Practices and Anthropometric Deficits Among Infants Attending Outpatient Services at an Urban Tertiary Care Hospital: A Cross-Sectional Study

**DOI:** 10.7759/cureus.84613

**Published:** 2025-05-22

**Authors:** Sara S Dhanawade, Sunil Kumar, Alka D Gore, Gracie Bhore

**Affiliations:** 1 Pediatrics, Bharati Vidyapeeth (Deemed to be University) Medical College and Hospital, Sangli, IND; 2 Pediatrics, ESIC Hospital Peenya, Bengaluru, IND; 3 Biostatistics, Bharati Vidyapeeth (Deemed to be University) Medical College and Hospital, Sangli, IND

**Keywords:** anthropometric deficits, breastfeeding, complementary feeding, dietary diversity, infants, nutritional status

## Abstract

Introduction

Improper infant feeding practices remain prevalent in developing countries, despite the existence of established guidelines. Gaining insights into these practices and their regional variations is essential for strengthening public health interventions.

Methods

This cross-sectional study involved 91 mother-infant pairs attending the outpatient services of an urban tertiary care hospital. Data were collected through interviews using a predesigned questionnaire. Feeding practices were assessed using a 24-hour dietary recall, and minimum meal frequency (MMF) and minimum dietary diversity (MDD) were documented. Statistical analysis included the chi-square test and unpaired t-test. ORs with 95% CIs were calculated, and binary logistic regression models were developed for wasting, stunting, and underweight as dependent variables. Data analysis was performed using IBM SPSS Statistics for Windows, Version 29.0 (Released 2022; IBM Corp., Armonk, NY, USA).

Results

The majority (88%) of mothers belonged to middle- or upper-income groups, and over half (57%) were graduates. Exclusive breastfeeding was reported in 59 (64.8%) cases, and complementary feeding was introduced at six months in 64 (70.3%) infants. However, solid foods were introduced between seven and 12 months in only 31 (34.1%) infants. Continued breastfeeding was observed in 19 (51.35%) infants aged six to eight months but declined to 13 (24%) among those aged nine to 12 months. MMF was adequate in only seven (16.21%) infants aged six to eight months and in 26 (44.44%) infants aged nine to 12 months. An MDD score above 4 was achieved in 52 (57.14%) infants. The prevalence of anthropometric deficits was as follows: underweight in 29 (31.87%), stunting in 24 (26.37%), and wasting in 11 (12.09%). Birth weight showed a significant association with all three anthropometric deficits (p < 0.01). Multivariate analysis identified age (six to eight months) and a dietary diversity score below 5 as significant factors for underweight, while only age remained significant for stunting.

Conclusions

Infant feeding practices were suboptimal despite the relatively favorable socioeconomic and educational status of the mothers, highlighting gaps in the implementation of infant and young child feeding guidelines. Continuous support from healthcare workers is essential to guide mothers and caregivers on appropriate infant nutrition and feeding practices.

## Introduction

Infant feeding practices have garnered the attention of researchers worldwide. Nutritional status during the first year of life plays a crucial role in determining a child’s growth, development, and overall survival. WHO recommends exclusive breastfeeding for the first six months of life, followed by the introduction of safe and appropriate complementary feeding (CF) up to two years of age [1]. The first two years are particularly critical due to the high nutritional demands required to support rapid growth and development. This period is thus highly susceptible to growth faltering, micronutrient deficiencies, and childhood illnesses.

CF is a key factor in supporting the optimal growth and development of infants. Around six months of age, breast milk alone becomes insufficient to meet an infant’s nutritional needs, increasing the risk of undernutrition. Therefore, the timely initiation of CF is essential. However, both early and delayed introduction of complementary foods can contribute to undernutrition. The timing and effectiveness of introducing solids are influenced by several factors, including household income, maternal age, residence, education level, number of children, and breastfeeding duration [2].

Introducing CF before six months can displace breast milk and increase the risk of infections such as diarrhea. Moreover, infants younger than six months may not be physiologically prepared for complementary foods due to the immaturity of their gastrointestinal tract, kidneys, and neurodevelopmental systems. The guiding principles of CF include exclusive breastfeeding until six months, initiation of CF at six months (180 days), and continuation of breastfeeding until two years of age. Additional principles involve responsive feeding, safe preparation and storage of complementary foods, ensuring adequacy in consistency, frequency, energy density, and nutrient content, as well as the use of vitamin and mineral supplements for infants and mothers. Feeding practices should also be adapted during and after illness [3-5].

The Lancet Child Survival Series identified CF as the third most effective intervention for reducing child mortality, with the potential to prevent 6% of under-five deaths [6]. Despite progress in tackling childhood undernutrition, the burden remains high in low- and middle-income countries (LMICs). Globally, 149 million children under five are stunted and 49 million are wasted, with LMICs accounting for 65% of stunted and 75% of wasted children. The majority of stunting occurs during the CF period in these countries [7-9].

According to the National Family Health Survey (NFHS-5, 2019-2020), only 11% of Indian children aged six to 23 months receive an adequate diet. Stunting and wasting are more common in rural areas than in urban ones [10]. The survey also reported that 35.8% of children under five are underweight, 38.4% are stunted, and 21% are wasted [11]. The success of CF depends heavily on access to accurate information and consistent support from trained individuals within families, communities, and the healthcare system.

Despite improvements in health services, inappropriate feeding practices remain widespread in Indian communities. Complementary foods are often introduced too early or too late, offered in insufficient quantities, or lack appropriate nutritional quality and frequency. Strengthening public health strategies requires a thorough understanding of current feeding practices.

This study aims to assess early infant feeding practices, their associated determinants, and nutritional status among infants attending an urban tertiary care hospital, with the following objectives: (1) to assess feeding practices among infants aged six to 12 months attending the outpatient department of an urban tertiary care hospital and (2) to evaluate the association between these feeding practices and anthropometric deficits.

## Materials and methods

A hospital-based, cross-sectional observational study was conducted among mothers and their infants aged six to 12 months who attended the pediatric outpatient department, well-baby clinic, and immunization clinic of a tertiary care hospital over a six-month period (April 2021 to September 2021). Mothers who were unwilling to provide consent, infants not accompanied by their mothers, and those with any illness that could affect feeding were excluded from the study.

The sample size was calculated using a power analysis for a one-sample proportion test conducted in IBM SPSS Statistics for Windows, Version 29.0 (Released 2022; IBM Corp., Armonk, NY, USA). Based on an expected prevalence of wasting at 43.7% [12], a minimum sample size of 72 participants was determined, with 80% power and a 5% level of significance (α = 0.05). A total of 91 participants were ultimately enrolled using a convenience sampling technique.

Data were collected by a pediatric resident (SK) under the supervision of a nutritionist (GB) with specialized expertise in child nutrition. Informed consent was obtained from each participant before data collection. A structured, pre-validated, and predesigned questionnaire consisting solely of closed-ended questions was administered verbally by trained interviewers. The questionnaire assessed adherence to recommended feeding practices, focusing on the duration of exclusive breastfeeding, age of initiation of CF, food consistency and adequacy, and the number of food items included. Open-ended responses regarding reasons for nonadherence were not collected. Sociodemographic information was also recorded.

Feeding practices were assessed using a 24-hour dietary recall. Minimum meal frequency (MMF) was defined as at least two meals per day for breastfed infants aged six to eight months and at least three meals per day for those aged nine to 12 months. For non-breastfed infants, a minimum of four meals per day was considered adequate. Minimum dietary diversity (MDD) was defined as the percentage of children over six months of age who consumed at least five of the defined food groups in the previous 24 hours [13,14].

Anthropometric measurements were recorded as follows: weight was measured using a digital weighing scale with a precision of ±5 grams, length was measured using an infantometer with a precision of 0.1 cm, and head circumference was measured using a non-stretchable measuring tape. Undernutrition was categorized according to WHO z-score classifications. An infant was considered wasted if the weight-for-length z-score was less than -2 SD, stunted if the length-for-age z-score was less than -2 SD, and underweight if the weight-for-age z-score was less than -2 SD. Scores between -2 and -3 SD indicated moderate undernutrition, while values below -3 SD indicated severe undernutrition.

For quantitative variables, means and SDs were calculated, while frequencies and percentages were used for qualitative variables. The chi-square test was used for univariate analysis to assess associations, and the unpaired t-test was applied to compare means between undernourished and normal infants. Binary logistic regression was used for multivariate analysis, with wasting, stunting, and underweight as dependent variables. Variables found to be significant in the univariate analysis were included as independent factors. A p-value < 0.05 was considered statistically significant, and p < 0.01 was considered highly significant. Data analysis was performed using Microsoft Excel (Microsoft Corporation, Redmond, WA, USA) and IBM SPSS Statistics for Windows, Version 29.0 (Released 2022; IBM Corp., Armonk, NY, USA).

The study received ethical approval from the Institutional Ethical Committee (BV(DU)MC&H/Sangli/IEC/428/21, dated February 2, 2021).

## Results

The study included 91 mother-infant pairs, with infants aged between six and 12 months, and an almost equal distribution of sex among participants. All 91 mothers (100%) reported receiving counseling on infant feeding practices.

Among the infants, 59 (64.8%) were exclusively breastfed for six months. Exclusive breastfeeding continued for only five months in 19 (20.9%) infants, while 13 (14.3%) continued exclusive breastfeeding until seven months. Breastfeeding continuation beyond six months declined significantly; while 19 (51.35%) infants aged six to eight months were still being breastfed, this number dropped to 13 (24%) among those aged nine to 12 months, a statistically significant decline (z = 2.68, p = 0.0074).

CF was initiated at six months in 64 (70.3%) infants, while 13 (14.3%) infants began CF at five months and 14 (15.4%) at seven months. MMF was found to be adequate (defined as three or more meals per day) in only six (16.21%) infants aged six to eight months, while 31 (83.79%) in this group received just one to two meals per day. Among infants aged nine to 12 months, 24 (44.44%) received adequate MMF, while 30 (55.55%) received less than the recommended number of meals per day.

Solid foods were introduced between seven and 11 months of age in only 31 (34.1%) infants, whereas 60 (65.9%) had not received solid foods even by 12 months of age. Sugar or salt was introduced at five months in 14 (15.4%) infants, at six months in 63 (69.2%) infants, and at seven months in another 14 (15.4%).

In terms of nutritional status, 11 (11.34%) infants were classified as wasted, 24 (24.74%) were stunted, and 29 (29.89%) were underweight. None of the infants showed severe wasting or stunting (defined as a z-score < -3 SD).

Table 1 presents the distribution of participants based on general characteristics. In total, 54 (59.3%) infants were in the nine- to 12-month age group. The gender distribution was nearly equal, with 46 (50.5%) females and 45 (49.5%) males. Most participants, 53 (58.2%), came from joint families, and 51 (56%) of the infants were second-born children. Regarding socioeconomic background, 43 (47.3%) belonged to middle-class families, and 52 (57.1%) of the mothers had an education level beyond graduation.

**Table 1:** General characteristics of study participants Socioeconomic status was classified according to the Modified Kuppuswamy Classification. LSCS, lower segment cesarean section; NVD, normal vaginal delivery

Variable	Number of participants	Percentage
Age group		
6-8 months	37	40.7
9-12 months	54	59.3
Sex		
Female	46	50.5
Male	45	49.5
NVD/LSCS		
LSCS	48	52.7
NVD	43	47.3
Type of family		
Joint	53	58.2
Nuclear	38	41.8
Birth order		
I	28	30.8
II	51	56
III	12	13.2
Socioeconomic class		
Lower class	11	12.1
Middle class	43	47.3
Upper class	37	40.7
Mother’s education		
Up to graduation	39	42.9
Graduation and above	52	57.1

Table 2 presents the univariate analysis comparing wasted and normal infants across various socio-demographic and related factors. Significant associations with wasting were observed for male sex (p = 0.001), nuclear family structure (p = 0.004), use of market-prepared complementary foods (p = 0.004), and a dietary diversity score below 4 (p = 0.033). However, these associations were not statistically significant in the multivariate analysis.

**Table 2:** Univariate analysis of wasted infants ^*^ Statistically significant at p < 0.05 ^**^ Highly significant at p < 0.01 To examine the association between wasting and sex, Fisher’s exact test was applied.

Factors	Wasting, n (%)	Normal, n (%)	Chi-square/Fisher’s exact	p-value
Age (months)				
6-8 months	7 (18.9%)	30 (81.1%)	2.738	0.098
9-12 months	4 (7.4%)	50 (92.6%)		
Sex^**^				
Male	11 (24.4%)	34 (75.6%)	-	0.001
Female	0 (0%)	46 (100%)		
Type of family^**^				
Joint	2 (3.8%)	51 (96.2%)	8.256	0.004
Nuclear	9 (23.7%)	29 (76.3%)		
Birth order				
I	4 (14.3%)	24 (85.7%)	0.614	0.736
II	5 (9.8%)	46 (90.2%)		
III	2 (16.7%)	10 (83.3%)		
Socioeconomic classes				
Lower	1 (9.1%)	10 (90.9%)	0.172	0.917
Middle	5 (11.6%)	38 (88.4%)		
Upper	5 (13.5%)	32 (86.5%)		
Mother’s education				
Up to graduation	5 (12.8%)	34 (87.2%)	0.034	0.853
Graduation and above	6 (11.5%)	46 (88.5%)		
Type of complementary food^**^				
Market food	9 (23.7%)	29 (76.3%)	8.256	0.004
Homemade food	2 (3.8%)	51 (96.2%)		
Adequate calories				
Less	10 (16.4%)	51 (83.6%)	3.228	0.072
Adequate	1 (3.3%)	29 (96.7%)		
Appropriate consistency				
No	9 (17.6%)	42 (82.4%)	3.374	0.066
Yes	2 (5.0%)	38 (95.0%)		
Dietary diversity score^*^				
<4	8 (20.5%)	31 (79.5%)	4.559	0.033
≥4	3 (5.8%)	49 (94.2%)		

Table 3 presents the univariate analysis of factors associated with stunting (height-for-age < -2 SD). Significant associations were found with the six- to eight-month age group (p < 0.001), male sex (p = 0.015), and a dietary diversity score below 4 (p = 0.023). However, in the multivariate analysis, only the six- to eight-month age group remained significantly associated with stunting (p < 0.001).

**Table 3:** Univariate analysis of stunted infants ^*^ Statistically significant at p < 0.05 ^**^ Highly significant at p < 0.01

Factors	Stunted, n (%)	Normal, n (%)	Chi-square	p-value
Age (months)^**^				
6-8 months	20 (54.1%)	17 (45.9%)	24.603	<0.001
9-12 months	4 (7.4%)	50 (92.6%)		
Sex^*^				
Male	17 (37.8%)	28 (62.2%)	5.962	0.015
Female	7 (15.2%)	39 (84.8%)		
Type of family				
Joint	10 (18.9%)	43 (81.1%)	3.682	0.055
Nuclear	14 (36.8%)	24 (63.2%)		
Birth order				
I	4 (14.3%)	24 (85.7%)	3.391	0.184
II	17 (33.3%)	34 (66.7%)		
III	3 (25.0%)	9 (75.0%)		
Socioeconomic classes				
Lower	3 (27.3%)	8 (72.7%)	0.434	0.805
Middle	10 (23.3%)	33(76.7%)		
Upper	11 (29.7%)	26 70.3%)		
Mother’s education				
Up to graduation	8 (20.5%)	31 (79.5%)	1.207	0.272
Graduation and above	16 (30.8%)	36 (69.2%)		
Type of complementary food				
Market food	21 (55.3%)	17 (44.7%)	3.682	0.055
Homemade food	8 (15.1%)	45 (84.9%)		
Adequate calories^*^				
Less	20 (32.8%)	41 (67.2%)	3.919	0.048
Adequate	4 (13.3%)	26 (86.7%)		
Appropriate consistency				
No	14 (27.5%)	37 (72.5%)	0.069	0.792
Yes	10 (25.0%)	30 (75.0%)		
Dietary diversity score^*^				
<4	15 (38.5%)	24 (61.5%)	5.136	0.023
≥4	9 (17.3%)	43 (82.7%)		

Table 4 presents the univariate analysis of factors associated with underweight status in children (weight-for-age < -2 SD).

**Table 4:** Univariate analysis of underweight infants ^* ^Statistically significant at p < 0.05 ^**^ Highly significant at p < 0.01

Factors	Underweight, n (%)	Normal, n (%)	Chi-square	p-value
Age (months)^**^				
6-8 months	21 (56.8%)	16 (43.2%)	17.789	<0.001
9-12 months	8 (14.8%)	46 (85.2%)		
Sex^**^				
Male	24 (53.3%)	21 (46.7%)	18.891	<0.001
Female	5 (10.9%)	41 (89.1%)		
Type of family^**^				
Joint	7 (13.2%)	46 (86.8%)	20.355	<0.001
Nuclear	22 (57.9%)	16 (42.1%)		
Birth order				
I	5 (17.9%)	23 (82.1%)	3.812	0.149
II	20 (39.2%)	31 (60.8%)		
III	4 (33.3%)	8 (66.7%)		
Socioeconomic classes				
Lower	5 (45.5%)	6 (54.5%)	1.066	0.587
Middle	13 (30.2%)	30 (69.8%)		
Upper	11 (29.7%)	26 (70.3%)		
Mother’s education				
Up to graduation	13 (33.3%)	26 (66.7%)	0.067	0.795
Graduation and above	16 (30.8%)	36 (69.2%)		
Type of complementary food^**^				
Market food	21 (55.3%)	17 (44.7%)	16.447	<0.001
Homemade food	8 (15.1%)	45 (84.9%)		
Adequate calories^**^				
Less	26 (42.6%)	35 (57.4%)	9.857	0.002
Adequate	3 (10.0%)	27 (90.0%)		
Appropriate consistency^**^				
No	23 (45.1%)	28 (54.9%)	9.353	0.002
Yes	6 (15.0%)	34 (85.0%)		
Dietary diversity score^**^				
<4	23 (59.0%)	16 (41.0%)	23.096	0.001
≥4	6 (11.5%)	46 (88.5%)		

Univariate analysis revealed significant associations with the six- to eight-month age group (p < 0.001), male sex (p < 0.001), nuclear family structure (p < 0.001), consumption of market-prepared complementary food (p < 0.001), inadequate caloric intake (p = 0.002), inappropriate food consistency (p = 0.002), and low dietary diversity (p < 0.001). Multivariate analysis showed that, after adjustment, only the six- to eight-month age group (OR: 0.36; 95% CI: 0.19-0.68; p = 0.002) and low dietary diversity (OR: 0.01; 95% CI: 0-0.23; p = 0.014) remained significantly associated with underweight status.

The findings in Table 5 indicate that infants classified as wasted, stunted, or underweight had significantly lower mean birth weights.

**Table 5:** Comparison of birth weight (kg) and nutritional status ^**^ Highly significant at p < 0.01

Nutritional status	Mean	SD	Unpaired t	p-value
Wasting^**^				
Yes (n= 11)	2.83	0.17	-3.496	0.002
No (n = 80)	3.04	0.31		
Stunting^**^				
Yes (n = 24)	2.86	0.29	-3.088	0.004
No (n = 67)	3.07	0.29		
Underweight^**^				
Yes (n = 29)	2.83	0.26	-4.608	<0.001
No (n = 62)	3.11	0.29		

## Discussion

We evaluated key indicators from the WHO-UNICEF IYCF guidelines, including the timely introduction of CF, MMF, MDD, and anthropometric deficits among infants attending outpatient and immunization clinics in an urban hospital.

In our study, 64.8% of infants were exclusively breastfed until six months, which aligns with the national average (64%) but is slightly lower than the average in Maharashtra (71%) [10]. Timely introduction of CF was observed in the majority (70.3%), whereas 15% experienced delayed introduction and 14% started early, both of which are undesirable practices but commonly seen. According to NFHS-5 data (2019-2020), only 42.7% of Indian infants aged six to eight months receive CF along with breast milk, with the figure rising to 52.7% in Maharashtra [10,14]. A 2023 study from Lucknow found that 50.3% of infants began CF at six months, with delays in 48% and early introduction in 1.7% [15]. Similarly, Rathaur et al. (2018) reported timely CF in 53.12% of infants [16]. In contrast, a hospital-based study from Central India showed that 84% of infants were started on CF between six and eight months [17]. These findings reflect considerable regional variation across India despite the existence of national guidelines. Therefore, assessing local feeding practices remains both relevant and essential. Our findings were more favorable than those reported in most of the above studies and the NFHS-5 data.

The majority (88%) of infants in our sample belonged to the middle socioeconomic class, and 57% of mothers had completed graduation or higher education. Despite these favorable demographics, infant feeding practices were suboptimal. Although CF was initiated at six months in most cases, solids or soft solids were not introduced until 12 months in 65.9% of infants, and the consistency of food was appropriate in only 37.36%. This aligns with the observation by Aggarwal et al. (2008), who reported thick consistency in only 38% of cases [18]. In contrast, a community-based study from rural Lucknow reported that 78% of infants aged six to eight months were given solid or semi-solid foods [15]. Other Indian studies have reported solid food introduction in 53-59% of children [16]. Globally, a study analyzing infant feeding practices across developing countries found that 52% of caregivers introduced solids or semi-solids between six and eight months, with rates ranging from 15% in Somalia to 95% in Belarus [19].

In our study, only 16.21% of infants aged six to eight months met the recommended MMF, though this increased to 44.44% in the nine- to 12-month age group. By comparison, studies from other regions in India reported significantly higher MMF rates of 72.3% and 86% [15,17]. Continued breastfeeding was observed in only 51.35% of six- to eight-month-old infants, dropping to 24% among nine- to 12-month-olds. This decline coincided with an increase in infants meeting MMF requirements.

Only 57.14% of infants achieved an MDD score above 4, consistent with findings from a hospital-based study in Central India [17]. Unhealthy feeding practices, such as early introduction of sugar and salt, were present in all infants studied. Despite claims from most mothers that they had received counseling on infant nutrition, IYCF practices remained suboptimal. This indicates persistent gaps in the implementation of IYCF guidelines, likely stemming from limited awareness or inadequate support from healthcare workers. There is a need for sustained, personalized guidance from healthcare providers to promote appropriate infant feeding.

In terms of anthropometric outcomes, 31.87% of infants were underweight, 26.37% were stunted, and 12.09% were wasted. Infants aged six to eight months had higher rates of wasting (18.9% vs. 7.4%), stunting (54.1% vs. 7.4%), and underweight status (56.8% vs. 14.8%) compared to those aged nine to 12 months. Chhabra et al. reported that wasting peaks in the six- to eight-month age group and declines with age, while stunting and underweight status tend to increase over time [12]. Conversely, a study from Uttarakhand found no significant differences in malnutrition across age groups or between sexes [16]. In our analysis, wasting, stunting, and underweight status were significantly associated with male sex. While factors such as nuclear family structure, use of market-prepared food, and inappropriate food consistency were significantly associated with undernutrition in univariate analysis, they were not significant in the multivariate analysis. Only age (six to eight months) remained significantly associated with stunting in the multivariate model, while both age and low dietary diversity were significantly associated with underweight status.

A significant association was also found between low birth weight and anthropometric deficits, consistent with earlier research [6,12]. Infants who were underweight, stunted, or wasted had significantly lower mean birth weights than those with normal growth. Although underweight was the most prevalent deficit in our sample, it is concerning that 24.74% of infants were already stunted by 12 months of age. A study from Indonesia highlighted early stunting during infancy, with small-for-gestational-age status as a major predictor [20]. While wasting is often viewed as a transient condition that may resolve, stunting is considered largely irreversible without substantial environmental change [21,22]. Longitudinal data from Malawi, South Africa, and Pakistan showed that 20% of children were stunted by three months of age, increasing to 49% by 24 months. It is frequently assumed that early wasting may lead to stunting over time; indeed, several studies have observed this pattern [7,20,21]. However, some research has found high levels of stunting in populations with little wasting [21]. Recent literature suggests that stunting and wasting may be parallel responses to undernutrition rather than sequential outcomes [17,21,23].

Children with low MMF are at greater risk of wasting, stunting, and underweight status. Similarly, low MDD is associated with increased risk of stunting [7,20]. In our study, low MMF was significantly linked to underweight status, and a dietary diversity score below 5 was significantly associated with wasting, stunting, and undernutrition in univariate analysis.

The CF period is a critical window of vulnerability to stunting, with lifelong consequences. Stunting can begin very early in infancy, underscoring the importance of interventions that promote proper feeding practices and enhance the nutritional quality of complementary foods.

The strengths of our study include face-to-face interviews that reduced response bias, a targeted sample of infants aged six to 12 months relevant for developmental analysis, and real-world data reflecting adherence to national feeding guidelines in an urban setting. The study also highlights ongoing implementation gaps that require programmatic attention.

However, several limitations should be acknowledged. The small sample size may limit generalizability. Our focus on infant feeding practices excluded older children, possibly narrowing the scope of findings. Being a hospital-based and convenience-sampled study, selection bias is likely. The use of a 24-hour dietary recall method may have introduced recall bias. We did not examine behavioral or cultural barriers to proper feeding, and the cross-sectional design limits causal interpretation. Potential confounders, such as the mother’s emotional state, employment status, the counselor’s competence, and the involvement of elders in caregiving, were not accounted for and may have influenced outcomes.

## Conclusions

Exclusive breastfeeding up to six months was observed in 64.8% of infants, and the majority (70.3%) were introduced to CF on time, in accordance with guidelines. However, a substantial proportion of infants still received inappropriate feeding. Despite relatively high maternal education levels and favorable socioeconomic status, overall feeding practices remained suboptimal. The introduction of solids or semisolids was delayed in many cases, and a significant number of mothers continued to offer thin feeds, thereby failing to meet the infants’ caloric needs. Other key indicators, such as MMF and MDD, were also low in most infants. Additionally, there was poor adherence to the recommended practice of continued breastfeeding, with a marked decline in breastfeeding rates after six months. Among the anthropometric deficits, underweight was the most common (29.89%), followed by stunting (24.74%) and wasting (11.34%). On multivariate analysis, after adjusting for confounders, age six to eight months remained significantly associated with stunting, while both age and dietary diversity score were significantly associated with underweight.

## Appendices

                                                                                    PROFORMA

ID no:

Age:                                                               Type of delivery: NVD/LSCS

Sex:                                                            

Place of delivery: Home/institutional birth weight (if known):                                                          Birth order:

Anthropometric data:

Weight:                                                                     MAC:

Height:                                                                      HC:

Weight for height:                                                    Height for age: Interpretation:

Sociodemographic data:

Maternal age:                                                   Family type: Joint/Nuclear

Mother's occupation:                                       Per capita income:

Mother's education:                                         Sociodemographic status: Upper class/Middle class/Higher class

Feeding practices:

1.  Was the child exclusively breastfed up to 6 months                         Yes/No

2.  Whether the mother still continues to breastfeed the child             Yes/No, Frequency of breastfeeding?

3.  Whether the child was given top feed                                               Yes/No, Artificial feeding/Mixed feeding/Bottle feeding

4.  Whether mother received any counselling regarding complementary feeding:           Yes/No Hospital visit for immunization/OPD visit/Elders of family

5. When was complimentary feeding started: <6 months/at 6 months/>6 months if delayed. Reason for delay

6. Type of complementary feed: Market food/Home-based food

7. Introduced to sugar/salt- Yes/No

At what age: <10 months/At 10 to 12 months/>12 months

8. When was solids introduced: 6 months/At 6 months/>6 months       Yes/No

**Table 6:** Dietary history

Food item	Quantity	No. of feeds	Quantity (gm/ml)	Consistency	Energy	Protein
					Total	

Impression:

(a) Adequate calories for age OR less for age

(b) Appropriate consistency: Yes/No

**Table 7:** Dietary diversity score

Sr. no.	Food groups	Examples	Yes = 1, No = 0
1.	Cereals	Rice, wheat, ragi, and maize	
2.	Nuts and legumes	Moong dal, urad dal, red gram dal, almond, cashew nut, and dates	
3.	Roots and tubers	Potato, carrot, and beetroot	
4.	Fruits	Mango, papaya, orange, chickoo, grapes, banana, and apple	
5.	Vegetables	Spinach and pumpkin	
6.	Diary products	Cow’s milk, buffalo milk, butter, cheese, curd, and paneer	
7.	Meat products	Egg, chicken, and fish	

Dietary diversity score: <4 (less than 4)/>4(more than 4)

Name:

Signature:
